# Effectiveness and Mechanism of Cryoablation in the Treatment of Oral Mucosal Melanoma

**DOI:** 10.1002/cam4.71577

**Published:** 2026-02-06

**Authors:** Zhu You, Tianqi Zhang, Li Dai, Jie Wen, Mingyang Liu, Tengda Zhao, Guozhu Yin, Yihua Wu, Shizhou Zhang

**Affiliations:** ^1^ Department of Oral and Maxillofacial Surgery Shandong Provincial Hospital Affiliated to Shandong First Medical University Jinan China; ^2^ Shandong First Medical University (Shandong Academy of Medical Sciences) Jinan China; ^3^ Department of Stomatology Shandong Provincial Hospital Affiliated to Shandong First Medical University Jinan China

**Keywords:** apoptosis, cryoablation, glycometabolism, immune microenvironment, oral mucosal melanoma

## Abstract

**Objectives:**

To assess the effectiveness and safety of cryoablation for oral mucosal melanoma (OMM) and explore its underlying mechanisms to provide insights for precision treatment of OMM.

**Materials and Methods:**

Patients diagnosed with OMM were divided into a cryoablation group and a noncryoablation therapy group. We compared the therapeutic outcomes of these groups and investigated the effects of cryoablation on glycometabolism, the immune microenvironment, and cell death modalities in the OMM.

**Results:**

The study included 32 OMM patients, with 18 in the cryoablation group and 14 in the noncryoablation therapy group. Cryoablation demonstrated high safety and effectiveness, with a postoperative survival rate of 72.22% (13/18). The median overall survival was 85.5 months (95% CI: 57.3–113.6) in the cryoablation group and 72.4 months (95% CI: 51.36–93.4) in the non‐cryoablation group. Significant changes in the immune microenvironment, including increased infiltration of CD4+, CD8+, and CD66+ cells and elevated expression of PD‐1, PD‐L1+, and CTLA4+ immune checkpoints, were observed postcryoablation. Conversely, FOXP3+ and CD19+ cell densities significantly decreased. Additionally, the expression levels of GLUT‐1, HIF‐1α, and PK‐M2 were notably reduced. The primary antitumor effect of cryoablation is attributed to apoptosis.

**Conclusion:**

Cryoablation is an effective treatment for OMM, and its antitumor effects are potentially linked to the modulation of the immune microenvironment, alteration of glucose metabolism, and induction of apoptosis.

## 1 Introduction

Oral mucosal melanoma (OMM) is a rare malignancy, representing only 0.5% of all oral cancers and 0.2%–8.0% of all melanomas (MM) [1]. The anatomical location of the OMM is relatively concealed and asymptomatic in its early stages, complicating early diagnosis and treatment. Compared with cutaneous melanoma (CM), OMM is more aggressive and has a poorer prognosis, with a 5‐year survival rate ranging from 15.0% to 57.4% and a 10‐year survival rate of only 15.40% [2, 3, 4]. The transformation of melanocytes into the OMM is influenced by various factors, yet the precise molecular pathogenesis remains unclear. The contributing factors may include ill‐fitting dentures, smoking, mechanical trauma, and family history. OMM differs from CM in several aspects, including its typical location, lack of clear risk factors, distinct mutation spectrum, and late diagnosis, all of which contribute to a poor prognosis. Increasing evidence indicates that OMM has significantly different molecular and genetic characteristics than CM does, highlighting the heterogeneity between the two and suggesting that findings from CM studies may not fully apply to OMM [5]. Treatment options for OMM primarily involve cryoablation, surgical resection, adjuvant therapy, and radiotherapy. Achieving an adequate surgical margin can be challenging in the confined oral cavity; however, the moist and smooth oral mucosa is particularly suitable for cryoablation, as melanocytes are highly sensitive to low temperatures. Cryoablation, which began in the mid‐19th century with the use of ice salt solutions to manage pain and control bleeding from skin tumors, now employs coolants such as nitrogen, nitrous oxide, or carbon dioxide. Melanocytes are effectively destroyed at temperatures between −7°C and −4°C, whereas normal skin and mucosal keratinocytes or fibroblasts can withstand temperatures as low as −35°C to −20°C. The moist and smooth oral mucosa further enhances the efficacy of cryoablation for melanomas in this area. Although cryoablation has proven therapeutic for oral mucosal melanomas, its mechanisms are not yet fully understood. Cryoablation induces cell death through various mechanisms, including physical damage to cell membranes, stress responses, necrosis, apoptotic cascades, microvascular destruction, and the production of antitumor antibodies, as well as immune activation mediated by cytotoxic T cells and natural killer cells [6, 7]. Destroyed tumor tissue presents antigens to phagocytes, which generate antibodies that target residual tumors, including tumor‐specific membrane proteins. These antibodies bind to residual tumor cell proteins, leading to macrophage and neutrophil chemotaxis, activating the host immune system, and reducing the amount of remaining tumor tissue and distant metastases (known as the cryoimmunological effect). Previous studies suggest that cryoablation may promote antitumor effects through several mechanisms: it destroys tumor cells, induces proapoptotic Fas receptors, activates apoptotic cascades involving caspases 3 and 9, destroys tumor blood vessels, releases tumor antigens to trigger both innate and adaptive immune responses, and exposes tumor‐related antigens to guide dendritic cells to the tumor site, stimulating their maturation and migration to lymph nodes to activate specific cellular immune responses. Although tumor‐infiltrating lymphocytes and other factors may contribute to a distal effect postcryoablation, this effect alone is insufficient to treat metastatic tumors [8]. The advent of immunotherapy, particularly immune checkpoint inhibitors, has enhanced the distal effect of cryoablation [9]. In China, despite over 40 years of cryoablation use for OMM, most of the literature consists of case reports, with few studies on the mechanism of cryoablation in OMM. The rarity of this disease and the lack of an OMM mouse cell line for immune function studies have left gaps in understanding the mechanisms of cryoablation, especially its impact on the immune microenvironment. Recent evidence suggests that cryoablation can improve immune function and significantly enhance tumor‐specific immune responses; however, comprehensive studies on its effects in OMM patients are lacking, impeding the application of cryoimmunotherapy in the OMM.

This study addresses the critical scientific question of “the effectiveness and mechanism of cryoablation in treating OMM” by employing multiple immunohistochemistry methods and other experimental techniques to investigate changes in glycometabolism, the immune microenvironment, and cell death modalities in the OMM before and after cryoablation. This study aims to identify key targets related to OMM immunotherapy, reveal the immune mechanisms underlying cryoablation, provide a theoretical foundation for the efficacy of combined cryoablation and immunotherapy, and propose new approaches for the clinical application of comprehensive OMM treatment based on cryoablation. Ultimately, this research aims to reduce the economic and disease burden on OMM patients and the broader population.

## 2 Materials and Methods

### 2.1 Patient Cohorts and Samples

A total of 32 patients with oral mucosal melanoma (OMM) diagnosed between January 2010 and June 2023 at Shandong Provincial Hospital Affiliated with Shandong First Medical University were included. Cohort A consisted of 18 patients who received cryoablation therapy, whereas Cohort B comprised 14 patients who underwent noncryoablation therapy. Group assignment was based on patient preference and clinical indications discussed by a multidisciplinary team. In the cryoablation group, patients received argon‐based cryoablation therapy (using the contact‐based liquid nitrogen technique) under general anesthesia. All cryoablation procedures in this study were performed using a contact‐based liquid nitrogen technique. In this approach, a cotton swab soaked in liquid nitrogen was applied directly to the tumor surface until the lesion turned white and hardened, indicating adequate tissue freezing. As the nitrogen evaporated, the applicator was promptly replaced with a freshly soaked swab to ensure continuous freezing. Each freeze cycle lasted approximately 5 min, followed by a passive thawing phase. The entire procedure was repeated for 2 to 3 cycles per treatment session. In the noncryoablation group, patients received conventional treatment including surgical resection with or without adjuvant radiotherapy or chemotherapy. Treatment choice was determined according to tumor size, location, operability, and patient preference. Details of individual treatments are summarized in Table S1. Clinicopathologic information, including age, sex, lesion location, histological type, and follow‐up data, was reviewed. All paraffin‐embedded blocks diagnosed with OMM were evaluated by two experienced pathologists via hematoxylin and eosin (H&E) and immunohistochemical staining. Due to the rarity of oral mucosal melanoma, rigorous selection criteria were applied to ensure the reliability and consistency of the samples. Specifically, only cases with both cryobiopsy specimens and corresponding postoperative surgical specimens were included to allow direct case matching. Cases prior to 2020 were excluded because, in most instances, cryoablation and surgical samples were not systematically preserved in a way that enabled accurate pre‐ and post‐treatment pairing. Moreover, all included samples were collected after 2021, with paraffin‐embedded tissues stored for no more than 5 years. To preserve antigenicity and meet the requirements of multiplex immunofluorescence analysis, tissue sections were freshly prepared and processed immediately upon sectioning. This study was designed as a retrospective analysis and received approval from the Ethical Committee of Shandong Provincial Hospital Affiliated with Shandong First Medical University (SWYX: No. 2023‐488). The statement that written informed consent was waived because the study analyzed retrospective data and involved minimal risk to participants.

### 2.2 Histopathological Features and Glucose Metabolism

When available, the sections were analyzed for specific markers and indices of glucose metabolism and glycometabolism in the OMM via H&E and immunohistochemistry (IHC), as listed in Table 1. IHC reactivity in tumor tissues was assessed via light microscopy. The positive rates of all the markers were analyzed. The labeling indices for Ki‐67 were calculated, and the mean values and standard deviations were determined. The Mann–Whitney *U* test was employed, with *p* values < 0.05 considered statistically significant.

**TABLE 1:** Positivity and dilution of diagnostic marker immunoexpression in the OMM.

Antibodies	Pretreatment	Dilution	Company	Positive *n*/(%)
HMB45	EDTA HIER	Oerating fluid	ZSGB‐Bio	100
Melan A	EDTA HIER	Oerating fluid	ZSGB‐Bio	100
Vimintin	EDTA HIER	Oerating fluid	ZSGB‐Bio	100
S‐100	EDTA HIER	Oerating fluid	ZSGB‐Bio	100

Abbreviation: HIER, heat‐induced epitope retrieval.

### 2.3 Multiplex Immunofluorescence Staining

Multiplex immunofluorescence staining was employed to examine the immune microenvironment and the mechanisms of cell death induced by cryoablation in the treatment of OMM. Fixed paraffin‐embedded tumor tissues were collected from OMM specimens before and after cryoablation, with each slide cut to a thickness of 4 μm. After being incubated at 63°C for 60 min, the slides were dewaxed at room temperature. Antigen retrieval was performed in EDTA solution (pH 9.0), followed by three washes with TBST buffer. Diluent/Block with Opal Antibody Diluent/Block (AKOYA, ARD1001EA) was added, and the mixture was incubated at room temperature for 15 min. The primary antibody against caspase 3 (Abcam, ab32351, 1:200 dilution) was added, and the samples were incubated at 37°C for 60 min and washed three times with TBST buffer. The secondary antibody enzyme‐labeled goat anti‐mouse/rabbit IgG polymer (ZSGB‐BIO, PV‐8000) was added, the mixture was incubated at 37°C for 10 min, and the mixture was washed three times with TBST buffer. The fluorescent dye Opal 620 (Akoya, NEL861001KT, 1:100 dilution) was added, and the mixture was incubated at room temperature for 5 min, followed by three washes with TBST buffer. The slides were then boiled in Tris‐EDTA buffer (pH 9.0) for epitope retrieval/microwave treatment, and the liquid was removed after cooling to room temperature. The resulting antibodies were labeled by repeating the above steps (antibodies and fluorescent dye information are provided in Table 3). After all the antibodies were labeled, microwave treatment with Tris‐EDTA buffer (pH 9.0) was used to remove the antibody‐TSA complex. TSA single‐stain slides were subjected to microwave treatment, counterstained with DAPI (AKOYA, FP1490) for 5 min, and then mounted in antifade medium (NobleRyder, 10,052). Panoramic scanning was performed via Tissue FAXS software (version SL‐7.1.120), and imaging analysis was conducted via StrataQuest software (version 7.1.129). Cell and tissue type identification and protein expression quantification were performed on panoramic images via TissueGnostics software (version 7.1.129). Intelligent algorithms segment all cells in the tissue area centered on the nucleus. Manual training and machine learning methods were used to identify tissue types, dividing the tissue into different regions, such as the tumor and stroma. Protein expression quantification was carried out by determining the average fluorescence threshold for each detection marker, thus identifying the number of positive cells marked by the marker. Positive cells were defined as those with immunofluorescence signals exceeding the threshold and exhibiting correct expression patterns. All analysis methods were saved within an algorithm to facilitate subsequent analysis of multiple original multispectral images from the same slide. Two experienced pathologists calculated the number, percentage, and density of positive cells in slides with more than six selected fields under ×200 magnification. For further analysis, the percentage of positive cells relative to all nucleated cells in the tumor nests, tumor stroma, and total areas from the selected fields was used. In brief, the infiltration levels of CD4+, CD8+, CD68+, FOXP3+, PD‐1 (Programmed Death‐1), PD‐L1+ (PD‐1 ligand), CD16+, CD66+, CTLA4+ (cytotoxic T‐lymphocyte antigen‐4), CD19+, caspase 3, and GASDERMIN are discussed in this research.

### 2.4 Statistical Analysis

Statistical analyses were performed via Graphpad prism software version 9.4, SPSS version 27.0 (SPSS, Chicago, IL, USA), Tissue FAXS software (version SL‐7.1.120), StrataQuest software (version 7.1.129), TissueGnostics software (version 7.1.129). Disease‐free survival (DFS) was assessed via Kaplan–Meier curves, and the log‐rank test was employed for group comparisons. The last follow‐up date was June 1, 2024. Logistic regression analysis was conducted to identify variables correlated with cryoablation efficiency, and Cox proportional hazards regression analysis was utilized to identify significant determinants for DFS. Comparisons of immune cell infiltration levels between the pre‐ and postcryoablation treatment groups were conducted via paired *t*‐tests, whereas comparisons between the pre‐ and postcryoablation treatment groups were performed via the Wilcoxon test. All tests were two‐sided, and *p* values < 0.05 were considered significant unless otherwise specified.

## 3 Results

### 3.1 Patient Cohort

**FIGURE 1:**
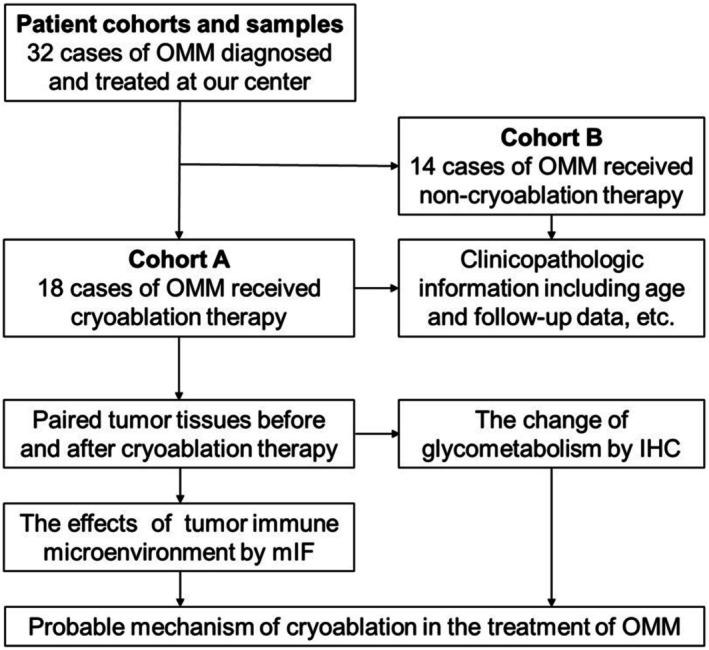
Flowchart of the study.

**FIGURE 2:**
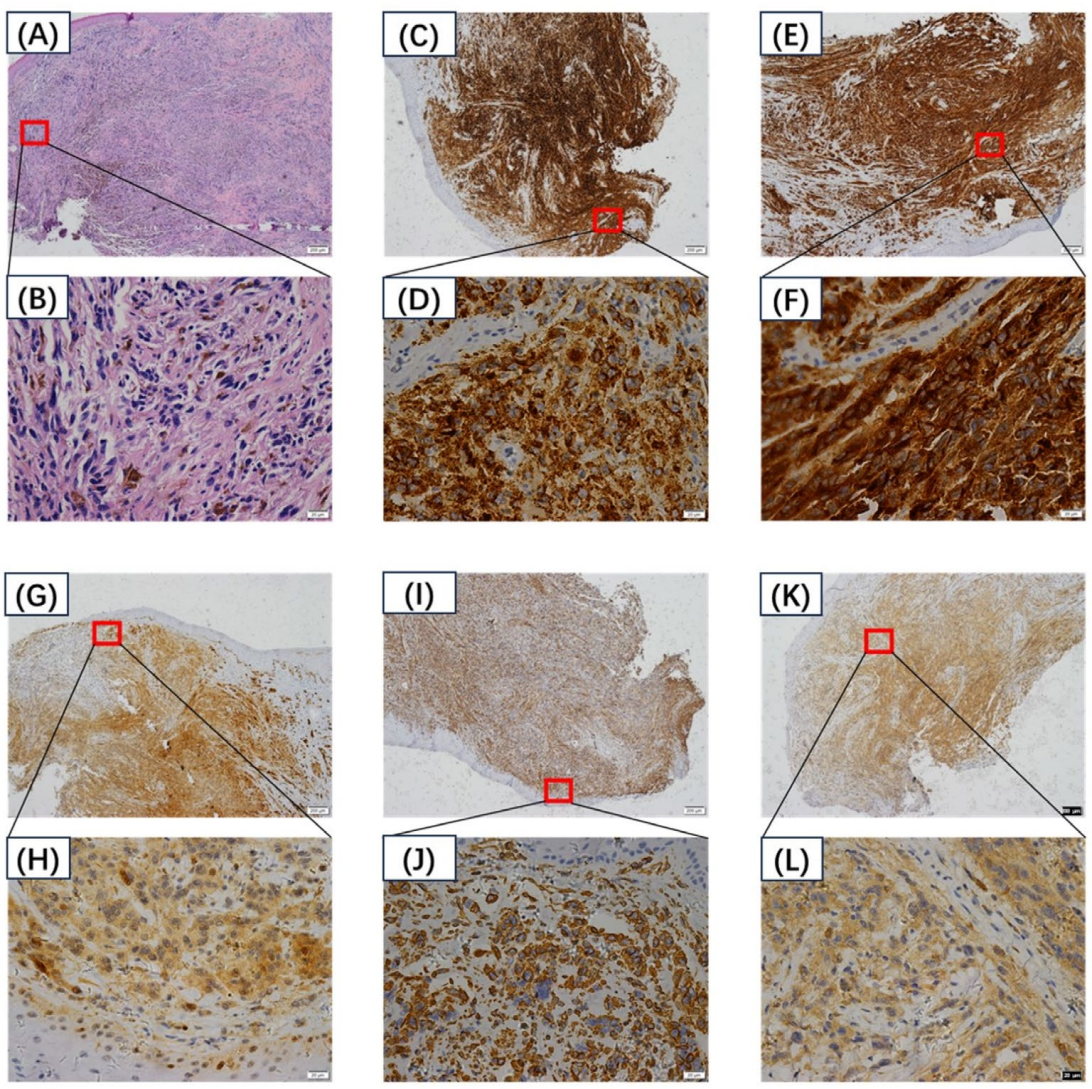
(A, B) Histopathological examination of OMM samples. (C, D) The reactions with HMB45 showed positive staining in all the samples. (E, F) The reactions with Melan A were positive in all the samples. (G, H) The reactions with Ki‐67 were negative in all the samples. (I, J) The reactions with Vimintin show negative staining in all the samples. (K, L) The reactions with S‐100 resulted in negative staining in all the samples.

This study included 32 patients who were diagnosed with OMM, flowchart of the study as shown in Figure 1. The accuracy of the diagnoses was confirmed through hematoxylin–eosin (H&E) staining and immunohistochemistry (IHC). Melanoma cells exhibited primarily dermatoid or spindle cell morphology, with occasional nevoid or plasma cell features. Cellular atypia, necrosis, and increased nuclear division indicate a high degree of malignancy. Spontaneous regression is characterized by fibrotic and granuloid hyperplasia, accompanied by scattered infiltration of lymphocytes, plasma cells, and phagocytic melanocytes. The OMM‐specific proteins HMB45 and Melan A presented 100% positive expression, as shown in Figure 2 and Table 1.

**FIGURE 3:**
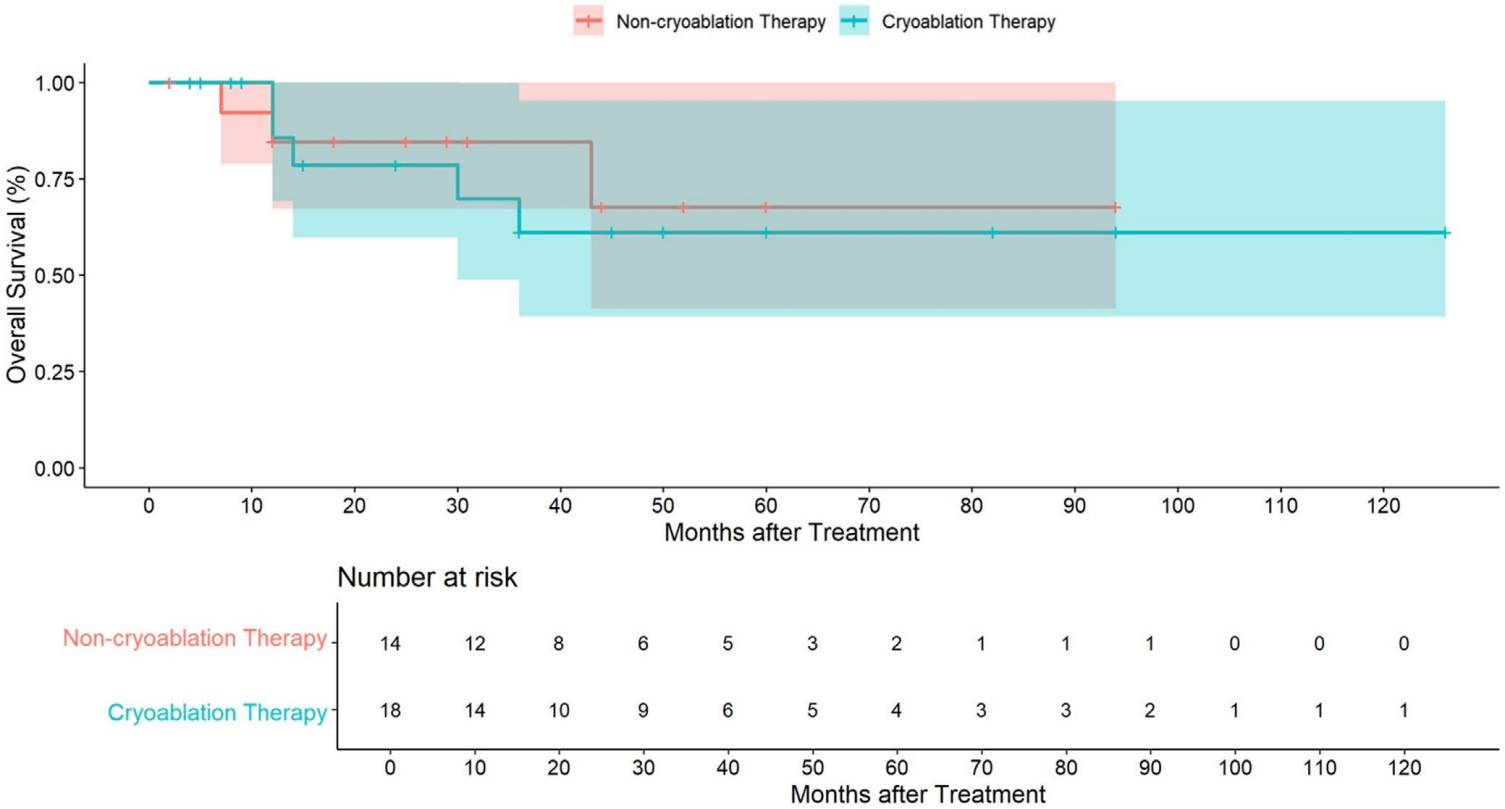
Overall survival (OS) graph.

Patients were divided into two cohorts on the basis of the treatment method: Cohort A (cryoablation group) and Cohort B (noncryoablation group). Cohort A comprised 18 patients, including 13 males and 5 females, with a mean age of 55.5 years. The cohort included 6 cases in the palate (33.3%), 6 cases in the gingiva (33.3%), 1 case in the cheek (5.6%), 1 case in the tongue (5.6%), 1 case in the lips (5.6%), and 3 cases in multiple body regions (16.7%). The recurrence rate was 55.6%, and the postoperative survival rate was 72.2%. Cohort B comprised 14 patients, including 5 males and 9 females, with a mean age of 59 years. The cohort included 4 cases in the palate (28.6%), 4 cases in the gingiva (28.6%), 1 case in the cheek (7.2%), 1 case in the tongue, and 4 cases in multiple regions (28.6%). The recurrence rate was 42.9%, and the postoperative survival rate was 78.6%, as detailed in Table 2 and Table S1. The median follow‐up period was 27.0 months (IQR 12.0–46.25). In the cryoablation therapy arm, the median follow‐up was 33.0 months (IQR 12.0–60.0), whereas in the noncryoablation therapy arm, it was 21.5 months (IQR 11.0–43.25). In total, 5 deaths were reported in the cryoablation therapy arm, and 3 were reported in the noncryoablation therapy arm. The median overall survival (OS) was 85.5 months (95% CI 57.3–113.6) for the cryoablation therapy arm and 72.4 months (95% CI 51.36–93.4) for the noncryoablation therapy arm, as detailed in Figure 3 and Table S1. Disease‐free survival (DFS) was analyzed to assess the therapeutic efficacy and disease control in both groups. The median DFS was 16.5 months in the cryoablation group versus 15.0 months in the non‐cryoablation group. Although the survival indicator appeared slightly longer in the cryoablation group, Kaplan–Meier analysis showed no statistically significant difference between the two treatment arms (log‐rank test, *p* > 0.05). These findings suggest that cryoablation therapy may achieve comparable disease control to conventional treatments for oral mucosal melanoma, while offering a minimally invasive alternative.

**TABLE 2:** Patient characteristics of Cohorts A and B.

Characteristics	Cohort A (*n* = 18)		Cohort B (*n* = 14)		*p*
	*n*	%	*n*	%	
Median age, years (range)	55.5 (41–86)		59 (50–74)		0.624
Gender					
Male	13	72.2%	5	35.7%	0.039
Female	5	27.8%	9	64.3%	
Lesion					
Palate	6	33.3%	4	28.6%	0.969
Gingiva	6	33.3%	4	28.6%	
Cheek	1	5.6%	1	7.2%	
Tongue	1	5.6%	1	7.2%	
Lips	1	5.6%	0	0	
Multiple	3	16.7%	4	28.6%	
Tumor relapse					
No	8	44.4%	8		0.476
Yes	10	55.6%	6	42.9%	
Vital status					
Alive	13	72.2%	11	78.6%	1.000
Dead	5	27.8%	3	21.4%	

#### 3.1.1 Reprogramming of Glucose Metabolism After Cryoablation for Mucosal Malignant Melanoma

**FIGURE 4:**
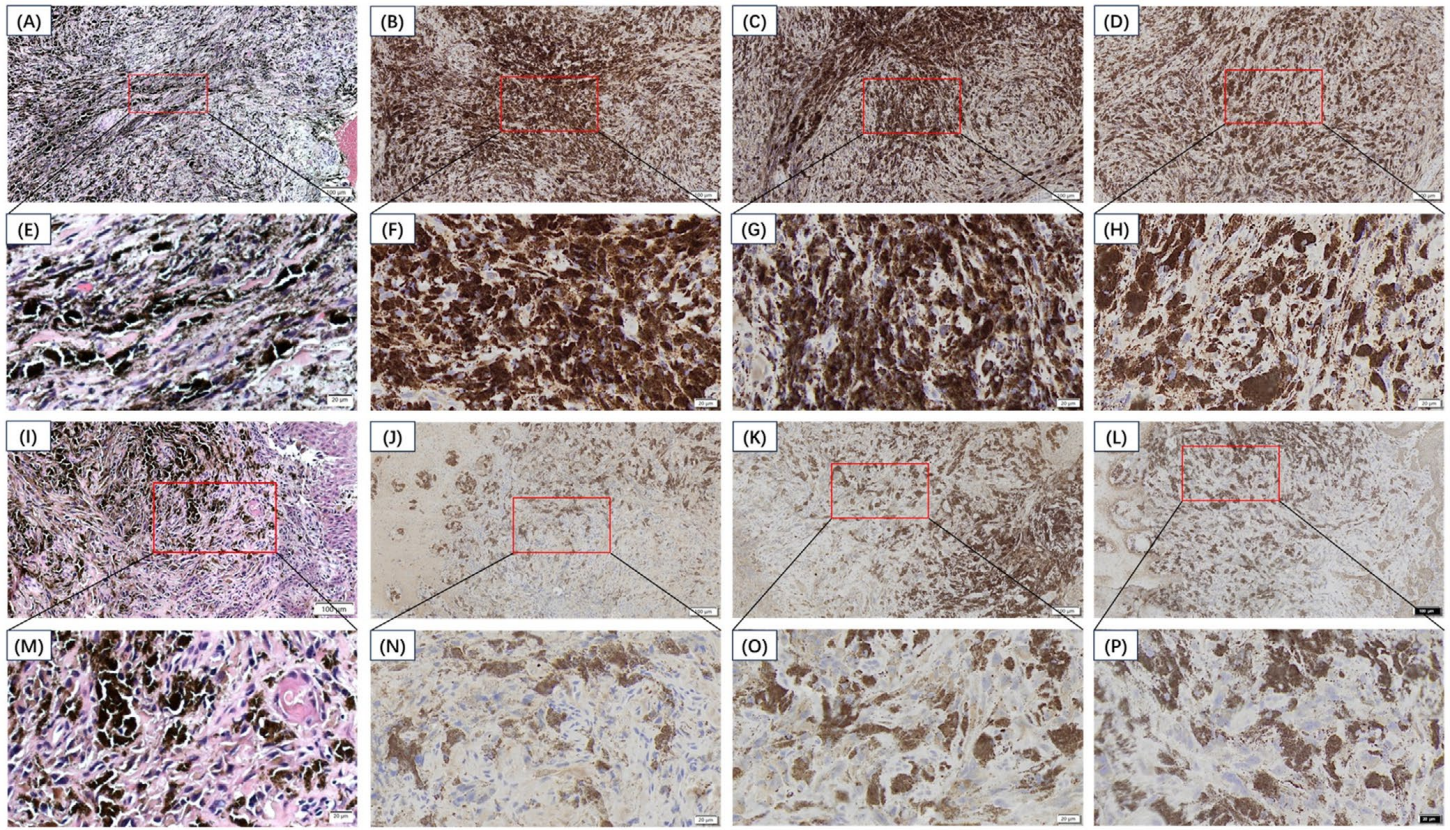
(A, E) Histopathological examination of precryoablation OMM cases. (B, F) The reactions with GLUT‐1 show strongly positive staining in precryoablation cases. (C, G) The reactions with HIF‐1α show strongly positive staining in precryoablation cases. (D, H) The reactions with PK‐M2 showed strongly positive staining in precryoablation cases. (I, M) Histopathological examination after cryoablation. (J, N) The reactions with GLUT‐1 showed weakly positive staining after cryoablation. (K, O) The reactions with HIF‐1α showed weakly positive staining after cryoablation. (L, P) The reactions with PK‐M2 show weakly positive staining after cryoablation.

H&E, GLUT‐1, HIF‐1α, and PK‐M2 staining before and after therapy is depicted in Figure 4. All three metabolism‐related proteins were expressed to varying degrees in all OMM patients before therapy. For GLUT‐1, 3 patients (75%) expressed GLUT‐1, and 1 patient (25%) expressed GLUT‐1 (+++). For HIF‐1α and PK‐M2, 2 patients (50%) had positive expression, and 2 patients (50%) had positive expression. The expression of these genes in patients with malignant melanoma was not significantly correlated with the demographic or clinical features of the OMM samples used in this study. After cryoablation, the expression levels of GLUT‐1, HIF‐1α, and PK‐M2 were significantly decreased. For GLUT‐1 and HIF‐1α, 1 patient (25%) had (−) expression, and 3 patients (75%) had (+) expression. For PK‐M2, 2 patients (50%) exhibited (−) expression, and 2 patients (50%) exhibited (+) expression. Detailed information is provided in Table 3.

**TABLE 3:** Positivity and dilution of metabolism‐related protein immunoexpression in the OMM before and after cryoablation.

Treatment	Antibodies	Pretreatment	Dilution	Company	−	+	++	+++
Before cryoablation	GLUT‐1	EDTA HIER	OF	Abcam	0	3	0	1
	HIF‐1α	EDTA HIER	OF	Abcam	0	2	2	0
	PK‐M2	EDTA HIER	OF	CST	0	2	2	0
After cryoablation	GLUT‐1	EDTA HIER	OF	Abcam	1	3	0	0
	HIF‐1α	EDTA HIER	OF	Abcam	1	3	0	0
	PK‐M2	EDTA HIER	OF	CST	2	2	0	0

Abbreviations: −, negative; +, weakly positive with < 33% of epithelial cells positive; ++, moderately positive with 33%–66% of epithelial cells positive; +++, strongly positive with more than 66% of epithelial cells positive; CST, Cell Signaling Technology; HIER, heat‐induced epitope retrieval; OF, operating fluid.

#### 3.1.2 Effects of Cryoablation on the Immune Microenvironment and Death Models of the OMM

**FIGURE 5:**
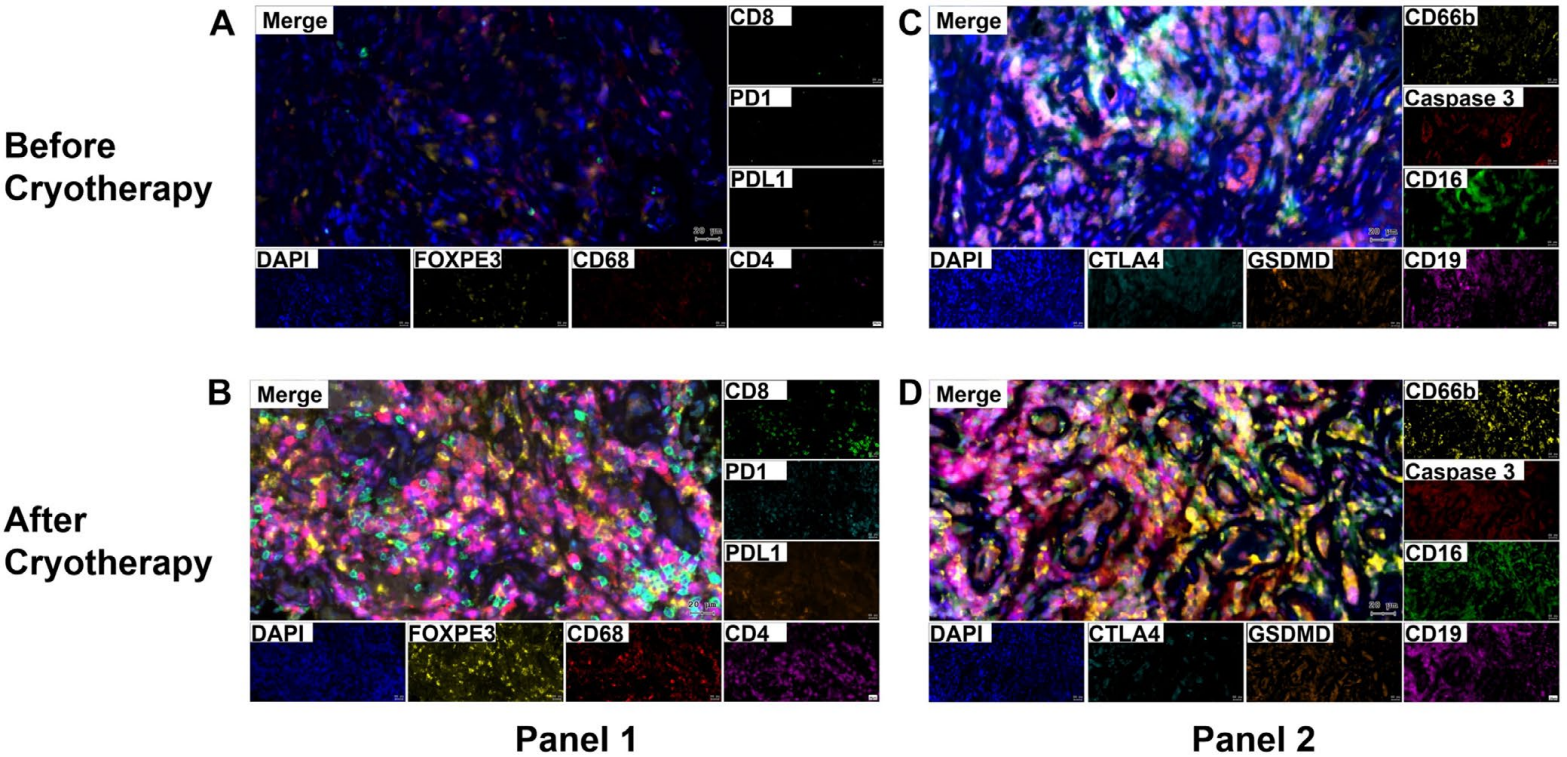
Multiplex immunofluorescence images of immune cell infiltration in pairs before cryoablation and after cryoablation of OMM specimens.

**FIGURE 6:**
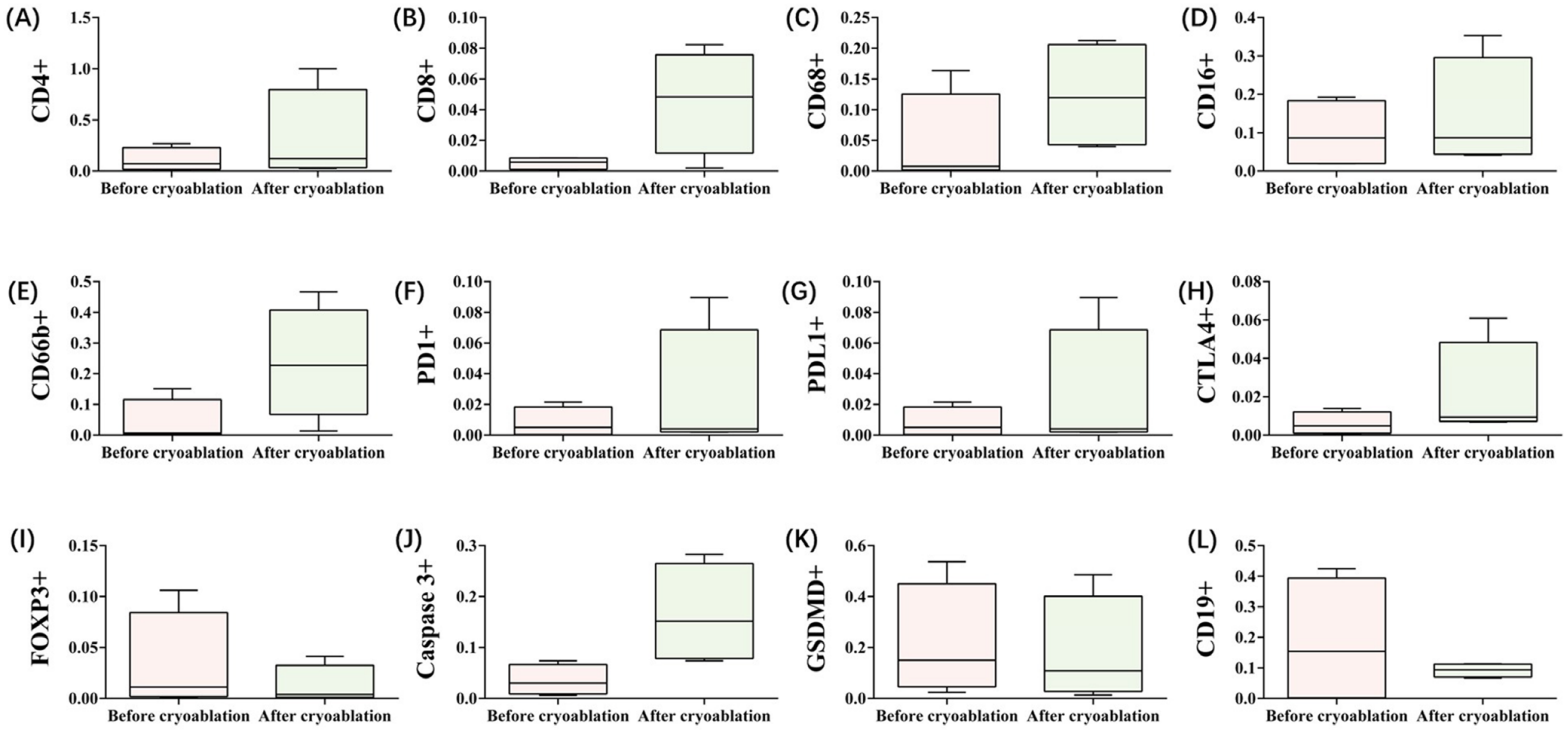
Comparison of total immune marker levels before and after cryoablation.

To analyze the tissue expression of CD4, CD8, CD68, FOXP3, PD‐1, PD‐L1, CD16, CD66, CTLA4, and CD19, samples from 4 patients (8 samples in total) were included. Biopsy and resection specimens from 4 patients in both the pre‐ and postcryoablation groups were analyzed. Representative histological slides stained before and after cryoablation are shown in Figure 3a,i. A greater density of cells expressing CD4, CD8, CD68, CD16, and CD66 was observed in tumor resections postcryoablation. The proportions of FOXP3+ and CD19+ cells exhibited different decreasing trends. With respect to changes in immune checkpoint proteins, the expression of PD‐1, PD‐L1, and CTLA4 increased. We further investigated models of cryoablation‐induced OMM cell death. The results indicated that the expression of caspase 3 was greater than that of GASDERMIN following cryoablation, as shown in Figure 5, Table S2, and Figure 6.

## 4 Discussion

Mucosal melanoma has a high degree of malignancy, and OMM is a rare malignant tumor that accounts for approximately 30% of head and neck mucosal melanomas and less than 1% of all melanomas in the whole body [10]. The incidence of OMM is closely related to age, with middle‐aged and elderly people being the most common patients, and the median age is approximately 55 years [11]. OMM has very strong metastasis ability and is the melanoma with the highest rate of cervical lymphatic metastasis, with a cervical lymphatic metastasis rate as high as 70% and a distant metastasis rate close to 40%, which is also the main reason for the poor prognosis of OMM [12].

OMMs originate from the neural crest and are located mostly near the basement membrane. Melanocytes become OMM due to various factors, but the molecular pathogenesis remains unclear, and the possible factors include poor dentures, smoking, mechanical trauma, and family history [1]. The molecular biological characteristics and gene profiles of OMM differ from those of cutaneous melanoma. Cutaneous melanoma is caused mainly by driver gene mutations induced by long‐term ultraviolet irradiation, mainly BRAF mutations. The most common gene mutation of OMM was KIT (23.1%), followed by NF1 (7.1%), RAS family (6.2%), and BRAF (3.1%) [13]. Another unique genetic characteristic of OMM is the high degree and structural variation of mutant genes, and approximately 85% of mucosal melanomas exhibit gene amplification [14]. Copy number variation was mainly concentrated in the amplification of TERT, CCND1, KIT, MITF, MDM2, CDK4, and NOTCH2, as well as in the copy deletions of NF1, PTEN, CDKN2A, ATM, and ARID1B. CDK4 amplification is most common in OMM, with CDK4 copy number amplification occurring in more than 50% of OMM [15].

The treatment of OMM mainly includes cryoablation, surgical treatment, adjuvant therapy and radiotherapy [1, 16]. The moist and smooth oral mucosa is an ideal site for cryoablation, and melanocytes are very sensitive to low temperatures. In China, cryoablation has been used for OMM for more than 40 years. The patellar OMM and some nodular OMM have a large range, are surrounded by a large number of satellite foci, and have limited anatomical space in the oral cavity. Therefore, it is difficult to obtain an ideal safe incisal margin through extended resection. Cryoablation can achieve a very good local control rate for OMM patients [17]. Although melanoma cells themselves are insensitive to radiotherapy and radiotherapy is not recommended for primary tumors, studies have shown that carbon ion radiotherapy with high bioefficiency may be a viable treatment for OMM [3]. The available evidence shows that mucosal melanoma has a low response to immunotherapeutic agents (such as immune checkpoint inhibitors such as ipilimumab and nivolumab and immunoproteasome inhibitors), and the efficacy of mucosal melanoma immunotherapy is mixed; thus, immunotherapy has not become a first‐line treatment for OMM [18, 19].

In this study, we assessed alterations in the immune microenvironment of patients with mucosal malignant melanoma before and after cryoablation. Despite the limited sample size, we observed an increase in the expression levels of various immune markers following cryoablation. CD4+ helper T cells play a crucial role in orchestrating the immune response. Their increase following cryotherapy suggests enhanced immune surveillance and the potential for a more effective antitumor response. The increased presence of CD4+ T cells can stimulate other immune cells, including CD8+ cytotoxic T cells, which are essential for targeting and eliminating tumor cells [20]. The observed increase in CD8+ cytotoxic T cells is particularly noteworthy, as these cells are directly responsible for tumor cell killing. This increase suggests that cryotherapy may elicit a more robust cytotoxic response, potentially leading to improved tumor control and eradication [21]. CD68, a marker for monocytes/macrophages, is often associated with inflammatory responses in the tumor microenvironment. Through rapid freezing and thawing, cryoablation induces tumor cell necrosis and apoptosis, resulting in the release of tumor antigens and cellular debris. These materials can activate macrophages, promoting their recruitment and accumulation at the tumor site, thereby enhancing the antitumor immune response. CD16, a marker for natural killer cells and certain macrophages, and CD66, associated with neutrophils, were also found to increase. These findings suggest that an enhanced innate immune response contributes to the immediate defense against tumor cells through mechanisms such as antibody‐dependent cellular cytotoxicity and neutrophil‐mediated cytotoxicity. The infiltration of several immune cells tended to decrease. FOXP3+ regulatory T cells (Tregs) are critical in the tumor microenvironment, as they suppress the function of effector T cells and promote tumor immune evasion [22]. The observed reduction in FOXP3+ Tregs following cryotherapy suggests that this treatment might enhance antitumor immune responses by decreasing the number of immunosuppressive cells, thereby inhibiting tumor growth. This finding is consistent with previous studies indicating that cryotherapy can disrupt tumor cells and release tumor antigens, thus activating the host immune system [23]. CD19+ B cells play a complex role in the tumor microenvironment, contributing to antitumor immunity through antibody production and antigen presentation, as well as tumor progression via immunosuppressive cytokine secretion [24]. The significant reduction in CD19+ B cells observed in this study may reflect a decrease in the number of tumor‐associated B cells that contribute to tumor immunosuppression. This reduction could further enhance antitumor immune responses. Conversely, we observed an increase in the expression levels of the immune checkpoints PD‐1, PD‐L1, and CTLA‐4 in oral mucosal melanoma (OMM) following cryotherapy. PD‐1 and PD‐L1 are critical immune checkpoint molecules. PD‐1 is expressed primarily on T cells, while PD‐L1 is expressed on numerous tumor cells and immune cells. They facilitate tumor evasion of immune surveillance by inhibiting T‐cell activation and proliferation. The increase in these molecules following cryotherapy may indicate an adaptive response of tumor cells to immune pressure [25, 26]. CTLA‐4 is another crucial negative regulatory molecule on T cells that inhibits T‐cell activation by binding to CD80/CD86. The increase in CTLA‐4 expression may suggest an active immune response in the local environment following cryotherapy, suggesting that tumors might be attempting to modulate the immune microenvironment. The heightened expression of immune checkpoints following cryotherapy could indicate that tumors may become more responsive to immune checkpoint inhibitors. Immune checkpoint inhibitors, including anti‐PD‐1, anti‐PD‐L1, and anti‐CTLA‐4 agents, function by blocking these negative regulatory pathways, thereby restoring immune attack against the tumor. Consequently, the increased expression of immune checkpoints following cryotherapy may augment the potential effectiveness of these inhibitors [27]. Combining cryotherapy with immune checkpoint inhibitors could improve therapeutic outcomes. Cryotherapy can induce tumor antigen release and enhance local immune responses, whereas immune checkpoint inhibitors can further mitigate immune suppression, resulting in more vigorous immune attack against the tumor [28]. Although combination therapy might improve treatment efficacy, it could also increase the risk of immune‐related adverse events. Thus, careful evaluation of the safety and tolerability of immune checkpoint inhibitors is essential in clinical applications. In this study, we observed a significant increase in the expression of the apoptosis marker caspase 3 in oral mucosal melanoma following cryoablation, whereas the expression of the pyroptosis‐related protein GASDERMIN remained unchanged. These findings offer valuable insights into the mechanisms underlying cryoablation in tumor treatment. Caspase 3 is a critical executioner enzyme in the apoptosis pathway, and its activation serves as a key indicator of apoptosis. Cryoablation induces cellular damage and death, resulting in an increase in apoptosis. Our results, which revealed a significant increase in caspase 3 expression, suggest that cryoablation effectively clears tumor cells via the apoptosis pathway. In contrast, GASDERMIN family proteins are associated with pyroptosis, a process related to inflammation. However, our study revealed no significant change in GASDERMIN expression, which may suggest that cryoablation has a limited effect on the pyroptosis pathway. Compared with pyroptosis, cryoablation primarily induces cell necrosis and apoptosis through abrupt temperature changes. Although we did not observe significant changes in GASDERMIN expression, these results may highlight the specificity of cryoablation for certain cell death pathways. GASDERMIN activation requires specific inflammatory stimuli and cellular signaling pathways. Cryoablation may not fully provide these conditions, or GASDERMIN expression may not be detectable within the short period following treatment. Although these observations did not achieve statistical significance, they elucidated various aspects of the impact of cryoablation on the tumor immune microenvironment. Future studies should involve larger sample sizes and further investigate the relationship between these immune changes and therapeutic outcomes to enhance the understanding and optimize the application of cryoablation in OMM.

## 5 Conclusions

In summary, our study demonstrated that cryoablation is both a safe and effective treatment for OMM. This intervention not only increases postoperative survival rates but also significantly affects the tumor immune microenvironment and glucose metabolism. Notably, we observed increased infiltration of immune cells (CD4+, CD8+, CD68+, CD16+, and CD66+) and elevated expression of immune checkpoints (PD‐1, PD‐L1+, and CTLA4+) following cryoablation. Furthermore, the observed reduction in FOXP3+ and CD19+ cell density suggests a shift toward a more robust antitumor immune response. The decreased expression of GLUT‐1, HIF‐1α, and PK‐M2 indicates a reprogramming of glucose metabolism, which may contribute to the observed antitumor effects. These findings support the potential of cryoablation as a precise therapeutic approach for OMM, which is predominantly driven by apoptosis‐induced cell death. Future research should address the limitations of the current study by incorporating larger, multicenter cohorts and extending the follow‐up period to evaluate long‐term efficacy and safety. Further experimental studies focusing on the molecular mechanisms of cryoablation are essential to elucidate the pathways through which it modulates the immune microenvironment and glucose metabolism. Additionally, exploring the combination of cryoablation with other therapeutic modalities, such as immunotherapy or targeted therapy, may enhance its antitumor efficacy. Ultimately, advancing our understanding of the mechanisms underlying cryoablation will facilitate its integration into precision medicine strategies for treating OMM and potentially other malignancies.

## 6 Limitations

Our study has several limitations. The relatively small sample size of 32 patients may limit the generalizability of our findings. Additionally, the limited follow‐up period raises uncertainties about long‐term outcomes. The observational nature of the study prevents the establishment of a definitive causal relationship between cryoablation and the observed changes in the immune microenvironment and glucose metabolism. Moreover, the molecular mechanisms underlying these changes require further investigation to fully elucidate the involved pathways.

## Author Contributions

**Zhu You:** data curation (equal), funding acquisition (lead), resources (lead), writing – original draft (lead). **Tianqi Zhang:** data curation (equal), formal analysis (lead), software (lead). **Li Dai:** investigation (lead), validation (equal), visualization (equal). **Jie Wen:** formal analysis (equal), investigation (equal), validation (equal). **Mingyang Liu:** methodology (equal), visualization (equal). **Tengda Zhao:** investigation (equal), validation (equal). **Guozhu Yin:** investigation (equal), methodology (equal). **Yihua Wu:** conceptualization (equal), project administration (lead), writing – review and editing (lead). **Shizhou Zhang:** conceptualization (lead), funding acquisition (lead), writing – review and editing (lead).

## Funding

This work was supported by the Shandong Provincial Natural Science Foundation (No. ZR2023QH376) and Health Commission of Shandong Province (No. 202408020583).

## Ethics Statement

This study was approved by the Ethical Committee of Shandong Provincial Hospital Affiliated with Shandong First Medical University (SWYX: No. 2023‐488).

## Conflicts of Interest

The authors declare no conflicts of interest.

## Supporting information


**Table S1:** Demographic, clinical, and pathological features of the studied samples.

**Table S2:** Changes of CD4, CD8, CD68, FOXP3, PD‐1, PD‐L1, CD16, CD66, CTLA4, and CD19 before and after cryotherapy in OMM.

## Data Availability

Data generated or used in this study are available from the corresponding authors upon reasonable request.
