# Supplementary material for: Effectiveness and Mechanism of Cryoablation in the Treatment of Oral Mucosal Melanoma
Source: Cancer Med. 2026 Feb 6;15(2):e71577. doi: 10.1002/cam4.71577 (PMC12881701; doi:10.1002/cam4.71577)
Supplement: Supplementary file 2 — Table S2: Changes of CD4, CD8, CD68, FOXP3, PD‐1, PD‐L1, CD16, CD66, CTLA4, and CD19 before and after cryotherapy in OMM. [file CAM4-15-e71577-s002.docx]

|  | Before cryoablation | After cryoablation | z | P value |
| --- | --- | --- | --- | --- |
| CD4+ | 0.071（0.016，0.229） | 0.121（0.035，0.795） | -1.461 | 0.144 |
| CD8+ | 0.006（0.001，0.009） | 0.048（0.012，0.076） | -1.461 | 0.144 |
| CD68+ | 0.008（0.002，0.125） | 0.12（0.043，0.206） | -1.826 | 0.068 |
| CD16+ | 0.087（0.02，0.183） | 0.087（0.044，0.296） | -1.095 | 0.273 |
| CD66b+ | 0.007（0.004，0.116） | 0.228（0.067，0.407） | -1.826 | 0.068 |
| PD1+ | 0.005（0.002，0.129） | 0.075（0.006，0.146） | -0.730 | 0.465 |
| PDL1+ | 0.005（0，0.018） | 0.004（0.002，0.069） | -0.730 | 0.465 |
| CTLA4+ | 0.005（0.001，0.012） | 0.009（0.007，0.048） | -1.826 | 0.068 |
| FOXP3+ | 0.002（0.001，0.004） | 0.011（0.002，0.084） | -1.342 | 0.18 |
| Caspase+ | 0.03（0.009，0.066） | 0.152（0.079，0.265） | -1.826 | 0.068 |
| GSDMD+ | 0.15（0.046，0.45） | 0.108（0.028，0.401） | -0.365 | 0.715 |
| CD19+ | 0.154（0.002，0.394） | 0.094（0.07，0.112） | -0.730 | 0.465 |

**Table S2** Changes of CD4, CD8, CD68, FOXP3, PD-1, PD-L1, CD16, CD66, CTLA4 and CD19 before and after cryotherapy in OMM
